# Stability in locomotive function of cilia according to clonal aging in *Paramecium*

**Published:** 2004-01-01

**Authors:** Satoshi Ohta, Nobuyuki Haga

**Affiliations:** Department of Biotechnology, Senshu University of Ishinomaki, 1, Shinmito, Minamisakai, Ishinomaki, Miyagi 986-8580

**Keywords:** Ciliary regeneration, locomotive function, clonal aging, *Paramecium*

## Abstract

Non-deteriorative cellular functions were examined in *Paramecium* and found in the cell lines undergoing proliferative senescence. Changes in two kinds of cellular functions, the ability to regenerate cilia and the locomotive function of cilia, were compared between young and old cells. The ability to regenerate cilia after artificial deciliation decreased in old *Paramecium* cells, but the ability of cilia to swim both forward and backward was stable with age. Our results suggest that morphogenesis of cilia is a deteriorative character, but the locomotive function of cilia is not associated with proliferative senescence.

## Introduction

The finite life span of proliferative cells is associated with the total number of cell divisions in an organism’s life history. Because each cell division involves the replication of DNA and all other cellular components, the proliferative life span is essentially associated with the replicative functions of the cell. The life-cycle phases of *Paramecium* have been defined as a function of the number of cell divisions after conjugation.1)–3) On the basis of the unique advantages of this organism, various kinds of molecules and cellular functions have been studied with respect to proliferative senescence.4) However, it remains unknown whether or not all cellular functions are committed to deteriorate with proliferative senescence.

During the course of seeking non-deteriorative cellular functions, we noticed that the locomotive function of cilia might be maintained until the end of the life span in cells of *Paramecium caudatum*. Cilia located on the ventral surface play an important role in sexual-cell recognition.5) The ability of cilia to recognize a complementary mating type and to initiate the reproductive process, called conjugation, is known to be a deteriorative character.6) On the other hand, cilia function as an electric scull when *Paramecium* swims. Ciliary movement is controlled by membrane potentials, various kinds of voltage-dependent ion channels and a dyninetublin motor system.7) While there have been extensive fundamental studies on the physiological, electrophysiological and biochemical properties of cilia, the role of aging on the locomotive function of cilia remains unknown.

In this study, we examined two kinds of ciliary functions, regenerative activity and locomotive activity, in young and old *Paramecium* cells. The important finding of our study is that although regenerative activity is associated with proliferative senescence, the locomotive activity of cilia is strongly maintained in old cells of *Paramecium*.

## Materials and methods

### Cells and culture method

All cells used in this study were *Paramecium caudatum*, syngen 3. Cells were grown at 23–25°C. The culture medium was 1.25% (w/v) fresh lettuce juice diluted with K-DS (Dryl’s solution modified by substituting KH_2_PO_4_ for NaH_2_PO_4_, pH 7.0 8)) and inoculated with *Klebsiella pneumoniae* one day before use.9)

The cell lines of both KNZ52 and SOS2 were established by conjugation and maintained in a continuous test-tube culture. The number of cell divisions after conjugation was estimated as previously described.10) Approximately 800 cells were transferred into a new test tube containing 2 ml fresh culture medium. The next day, 4 ml of fresh culture medium was added to each tube, followed by 8 ml on each of the next 2 days. The number of fissions between transfers was estimated, based on the number of cells in the first inoculums and the total number of cells by the fifth day. Under our experimental conditions, the total number of fissions in a single-tube culture was found to be approximately five.

### Deciliation and regeneration of cilia

A cell suspension was mixed with the same volume of 10% ethanol in K-DS. After standing for 10 min at 25°C, deciliation was performed by vortexing for 10–20 sec. Cells were washed with K-DS 3 times, and then immobilized cells were isolated into a depression-slide glass containing K-DS. The regeneration of cilia was induced in K-DS at 25°C.

### Measurement of the length of regenerating cilia

After deciliation, cells of KNZ52 (30 fission after conjugation) were periodically isolated, dried on a slide glass and photographed with a digital camera under a differential microscope. Photographs were processed in a personal computer and the length of a cilium was measured on the monitor. Cilia were measured from three cell surfaces (middle of dorsal surface and anteriorand posterior of ventral surfaces). The mean cilium length and standard deviation were calculated from 3 cells.

### Measurement of swimming speed

The swimming speed of cells (n = 10) was measured in a plastic dish, containing K-DS, marked with a 2 mm grid. The time for swimming across a 2 mm distance was measured with a stopwatch.

### Measurement of the duration of backward swimming

The tested cells (n = 10) were incubated in a standing solution containing 4 mM KCl, 1 mM CaCl_2_ and 1 mM Tris-HCl (pH 7.5) for 15 min and then transferred to a high potassium test solution containing 80 mM KCl, 1 mM CaCl_2_ and 1 mM Tris-HCl (pH 7.5). The duration of backward swimming, induced by the high potassium ion solution, was measured with a stopwatch under a binocular microscope.

## Results

### Comparison of ciliary regeneration between young and old Paramecium

**Fig. 1:**
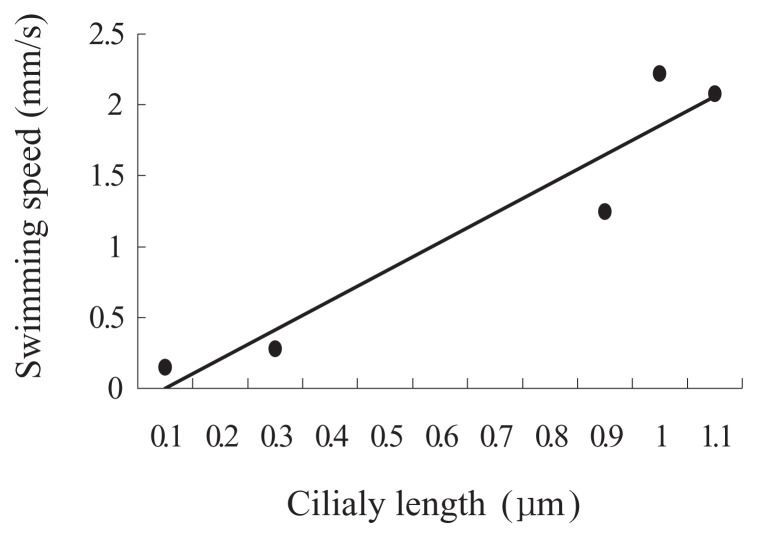
Correlation between the length of regenerating cilia and swimming speed of cells. After deciliation treatment, the swimming speed of cells was measured at the time indicated. The cells were then dried on a slide glass and photographed to measure the length of their cilia. Abscissa shows the time after deciliation and the ordinate swimming speed. The data points are the means of 10 individual cells (the standard deviations were not shown). The regression coefficient is 0.955 (p < 0.05).

**Fig. 2:**
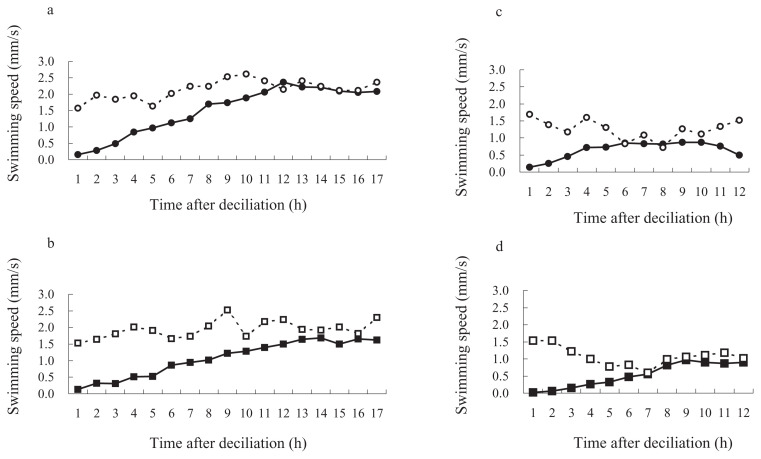
Time course of the recovery of swimming speed of deciliated cells. The swimming speed of deciliated cells was measured hourly after deciliation treatment. a, cells of KNZ52 at about 30 fissions after conjugation, b, at about 700 fissions after conjugation, c, the cells of SOS2 at about 50 fissions after conjugation and d, at about 500 fissions after conjugation. Closed circle or quadrilateral indicate deciliated cells and opened circle or quadrilateral indicate untreated control. Abscissa shows time after deciliation and ordinate swimming speed (n = 10).

**Fig. 3:**
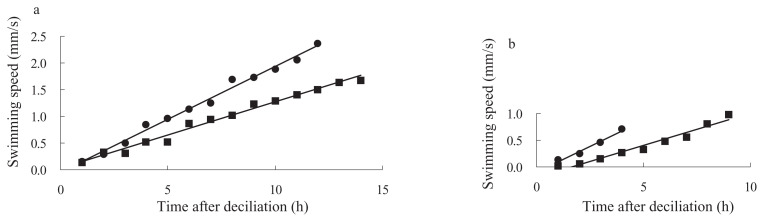
Regressions between ciliary regeneration and swimming speed. a, the data of KNZ52 from Fig. 2a (from 1 to 12 hours) and b (from 1 to 14 hours) were regressed (r for both 30 and 700 fissions were 0.990). b, the data of SOS2 from Fig. 2c (from 1 to 4 hours) and d (from 1 to 9 hours) were regressed (r for 50 fissions was 0.990 and for 500 was 0.980). Closed circles indicate young cells and closed quadrilaterals indicate old cells. The difference of slopes between young and old cells was statistically significant in both KNZ and SOS2 lines (p < 0.05). Abscissa shows time after deciliation and ordinate swimming speed.

Clonal ages of the cells used in this experiment were about 30 and 700 fissions after conjugation in KNZ52, and about 50 and 500 fissions after conjugation in SOS2. To test the ability of ciliary regeneration in old *Paramecium*, the cells of KNZ52 at about 30 and 700 fissions after conjugation were artificially deciliated using the ethanol method. After deciliation, immobilized cells were isolated into K-DS with a micropipette, under a binocular microscope, and incubated at 25°C. First, we examined the correlation between the length of regenerating cilia and swimming speed, to investigate the time course for recovery of swimming speed. As shown in Fig. 1, there was a statistically strong correlation between the length of cilia and swimming speed of cell (regression coefficient r = 0.955, p < 0.05, n = 10). Then, we used swimming speed as a characteristic for the regeneration of cilia. The swimming speed of cells was measured under a binocular microscope with a stopwatch at various times after deciliation. As shown in Fig. 2a and 2b, both young (30 fissions after conjugation) and old (700 fissions) cells continuously increased in swimming speed and reached their maximum in about 12 hours for the former and about 14 hours for the latter. The increase in swimming speed was analyzed by plotting the data of Fig. 2 into scatter diagrams (Fig. 3a, b). The regression lines of young and old *Paramecium* showed that the regression coefficients were highly correlated (Fig. 3). The comparison of the slopes of lines for young and old cells indicated that they were also statistically significant (student t-test, p < 0.05) (Fig. 3a).

To examine whether the observed reduction in the rate of ciliary regeneration in old cells of KNZ52 is a general character or not, we used a SOS2 cell line which has a different genetic background to KNZ52. As shown in Fig. 2c and 2d, the young cells (50 fissions after conjugation) of SOS2 reached their maximum level at about 4 hours and the old cells (500 fissions) reached it at about 9 hours. The regression lines of young and old cells showed that the regression coefficients were highly correlated (Fig. 3). The increasing swimming speed of old cells was lower than that of young cells and the difference of the slopes between the two lines was significant (student t-test, p < 0.05) (Fig. 3b).

### Locomotive function of cilia after ciliary regeneration

To qualify the function of regenerated cilia in both young and old *Paramecium*, the swimming speed of cells was measured at 24 hours after deciliation. As shown in Table I, there were no significant differences between before deciliation and after regeneration in all cell lines examined (basic t-test, p < 0.05).

### Ability to swim backward in a high potassium solution

*Paramecium* cells show backward swimming when stimulated. The backward swimming was caused by the change in direction of ciliary beating. This ciliary reversal is induced by membrane excitation, followed by the inward flow of calcium ions through Ca^++^-channels located in the ciliary membrane.11),12) We examined whether these complex processes involved in backward swimming function normally in old *Paramecium*. To quantify the ability to swim backwards, we used a high potassium test solution. After 15 min incubation in the standing solution, cells were transferred into the high potassium test solution, and we measured the time of continuous backward swimming with a stopwatch. As shown in Table II, there were no significant differences between young and old *Paramecium* in both the KNZ52 and SOS2 cell lines (basic t-test, p < 0.05).

## Discussion

Cilia have various kinds of important functions in the cells of *Paramecium*, such as sexual-cell recognition in conjugation, food uptake and cell locomotion. The expression of sexual activity, called mating reactivity, has been found to be deteriorative in clonal aging.6) As demonstrated in this report, a comparison of the ability to regenerate cilia and the locomotive function of regenerated cilia between young and old *Paramecium* cells revealed that the time course of ciliary regeneration was affected by aging, but the ability to swim forward was not. This indicates that the process necessary for the construction of cilia is a deteriorative character, but the function of reconstructed cilia is not, suggesting that the locomotive function of cilia is not associated with proliferative senescence.

Combined studies of genetic, biochemical and electrophysiological analyses have demonstrated that there are at least 7 complementation groups in the mutant of Ca^++^-channel functions in two species of *P. caudatum* and *P. tetraurelia*, indicating that at least 7 different types of genes are involved in the cells’ ability to swim backward.13) Our results indicate that cilia of old *Paramecium* work normally, as in those of young cells. The maintenance of the ability to swim backward in old *Paramecium* suggests two possibilities. One is that all genes responsible for the generation of backward swimming act normally. The other is that functional compensation for altered gene actions are involved.

It has been demonstrated that the life-cycle phases of *Paramecium*, such as sexual immaturity, maturity and senescence, are dependent on the number of cell divisions after conjugation,1)–3),14),15) and the deterioration of proliferative activity is associated with the total number of cell divisions after conjugation. The finding that cilia have stable locomotive function in old *Paramecium* cells presents a new point to be considered in the causal relationship between proliferative senescence and organelle functions. In this regard, the analysis of mechanisms for the maintenance of ciliary functions could give important clues for understanding the underlying molecular mechanisms in the cause of proliferative senescence.

## Figures and Tables

**Table I:** Recovery of the locomotive function of deciliated cells at 24 hours after deciliation

Cell lines	Clonal age	Swimming speed (mm/sec)	

		Deciliated	Control
KNZ52	f = 30	2.1 ± 0.4	1.8 ± 0.4
	f = 700	2.0 ± 0.3	1.8 ± 0.4
SOS2	f = 50	1.6 ± 0.5	1.9 ± 0.7
	f = 500	1.2 ± 0.3	1.4 ± 0.2

In all cell lines, there were no significant differences between “Deciliated” and “Control” (basic t-test, p < 0.05). f indicates fissions after conjugation. The values are mean ± SD (n = 10).

**Table II:** The ability to swim backward in the high potassium test solution

Cell lines	Clonal age	Duration of backward swimming (sec)
KNZ52	f = 30	71.9 ± 27.2
	f = 700	76.1 ± 26.6
SOS2	f = 50	59.9 ± 16.3
	f = 500	65.0 ± 21.9

Duration of backward swimming, measured with a stopwatch, in the test solution containing 80 mM KCl. There were no significant differences between young and old cell lines in both KNZ and SOS2 (basic t-test, p < 0.05). f indicates fissions after conjugation. The values are mean ± SD (n = 10).
